# Investigating multidimensional organisational trust through breach

**DOI:** 10.1080/00049530.2023.2260498

**Published:** 2023-10-04

**Authors:** Sarah Fischer, Julian Clarke, Arlene Walker, Shannon Hyder

**Affiliations:** School of Psychology, Deakin University, Geelong, Victoria, Australia

**Keywords:** Trust, psychological contract, psychological contract breach, psychological contract violation, leadership

## Abstract

**Objective:**

The literature shows employee trust in leaders supports organisational performance, despite much still being unknown about the employee-leader trust relationship. This study aimed to explore employee trust in leaders through trust breaches to broaden knowledge about the multidimensional nature of the trust construct and provide organisations with a focus on how to improve employee trust in leaders.

**Method:**

Trust breaches were explored using hypothetical vignettes. Participants received one scenario with a combination of a relationship treatment and behavioural treatment. There were three relationship treatments: no relationship, new relationship, and established relationship between employee and leader. There were also eight unique behavioural treatments of trust breach between employee and leader in the workplace (for example, betraying agreements).

**Results:**

Using one-way and two-way analysis of variance, findings showed that only those allocated to the established relationship group were likely to trust the hypothetical leader in the future. While all behavioural breaches negatively influenced future trust, the behaviours of leader lying to, betraying confidentiality of, and publicly belittling the employee were significantly less likely to engender future employee trust.

**Conclusion:**

This study offers a novel perspective to exploring employee-leader multidimensional organisational trust by investigating trust breaches according to type of relationship and behavioural breach type. This study showed behavioural breaches negatively influence perceptions of future employee trust in leaders. It also reaffirms that established relationships are most likely to be resilient to trust breaches over nominal or absent relationships between employees and their leader.

## Key Points

**What is already known about this topic:**

1. Employee trust in leaders has been considered a unidimensional and multidimensional construct.
2. Employee trust in leaders is essential for organisational performance.
3. Trust is hard to rebuild after a trust breach.

**What this topic adds:**

1. Trust breaches, like trust itself, have relational and behavioural nuances.
2. Behavioural trust breaches negatively influence likelihood the employee will trust the leader in the future.
3. The relationship between employee and leader determines the likelihood the employee will trust the leader in the future.

## Introduction

“People leave managers, not jobs” is a common phrase (Elzinga, 2023; Kelly, 2022; Saiz, 2022). Whilst this is anecdotal, there is also much literature on the topic of leader influence on poor wellbeing (Stengård et al., 2021) and intention to quit (Haque et al., 2019; Simard & Parent-Lamarche, 2022). Intention to quit and turnover have measurable negative effects on organisational performance (Haque, 2021). Equally, trust at work (also referred to as organisational trust) has a key role in supporting organisational performance and outcomes (Holtz et al., 2020; Zhao et al., 2007). When trust at work is breached, it can impact organisational commitment, job satisfaction and retention (Coyle-Shapiro et al., 2019). Most research regarding breach of trust in the workplace has concentrated on its effect on organisational outcomes (Coyle-Shapiro et al., 2019). Organisational trust can occur in a variety of contexts (Gillespie et al., 2021). There are group-level dynamics such as employee trust in the organisation or teams trusting teams where there are interdependencies. It can also occur on individual levels, such as between colleagues and between the employee and the leader. Whilst all contexts are worth exploration to understand the mechanisms that build and sustain trust in the workplace, the context encompassing employee trust in leader is the focus of this study.

### Employee trust in leaders

There may still be uncertainty regarding whether employee trust in leaders is a unidimensional or multidimensional construct (Dirks & Ferrin, 2002; McEvily & Tortoriello, 2011), but the evidence is growing that reinforces the multidimensionality of employee trust in leaders (Fischer et al., 2020, 2023; Iqbal et al., 2019; Saleem et al., 2020). The literature that discusses multidimensional organisational trust articulates the dimensions as behavioural and relational (Dirks & Ferrin, 2002; Fischer et al., 2020, 2023; McAllister, 1995). This research adds to the literature by examining trust breaches in the context of multidimensional organisational trust and explores whether trust is most influenced by the type of behaviour that caused the breach, by the relationship between employee and leader when breach occurs, or some combination of the two.

### Future employee trust in leaders

The literature above describes trust in the present. However, this research is focused on the potential for a breach to influence future employee trust in leaders. Possibly, the closest construct is trust propensity. Trust propensity reflects a person’s general willingness to trust others (Mayer et al., 1995; Rotter, 1967) and is viewed as dispositional, or an individual trait (Heyns & Rothmann, 2015), though a recent study suggests trust propensity is not always stable and can have state-like qualities (Baer et al., 2018). Those high in trust propensity are more likely to be willing to be vulnerable to others and to trust them (Alarcon et al., 2016). Trust propensity appears to predict trust independently from other factors of perceived trustworthiness (Colquitt et al., 2007) and is a significant predictor of employee trust in their employer (Searle et al., 2011).

The employee–leader relationship when the breach occurred is important to consider, and therefore there are some challenges with the construct of trust propensity. The influence of trust propensity on employee-leader trust may be affected by the length of the relationship between employee and leader (Mayer et al., 1995; McKnight et al., 1998). Trust propensity is particularly important in new relationships, such as where an employee and leader are working together for the first time (McKnight et al., 1998; Rotter, 1967). In this circumstance, employees have little or no information about their leader and so make assessments of trust based on their trust propensity disposition (Schoorman et al., 2007). Trust propensity is less important in established relationships, where an employee and leader are familiar with each other (Alarcon et al., 2016). As the amount of information an employee has about their leader increases, employees move from a rational choice to an information processing model (Jones & Shah, 2016). Assessments about whether to trust a leader become less based on employees’ trust propensity disposition and more on their experience of the leader’s trustworthiness (Schoorman et al., 2007). On balance, the research suggests that the influence of trust propensity on intention to trust diminishes over time (Alarcon et al., 2016) as the length of the trustor–trustee relationship increases and the trustor becomes more familiar with the trustee based on actual experience (Levin et al., 2004).

### Trust breaches in the workplace

As employee trust in leaders has a behavioural and relational element (Dirks & Ferrin, 2002; Fischer et al., 2020, 2023; Schoorman et al., 2007), trust breaches may also have behavioural or relational nuances (Morrison & Robinson, 1997; Robinson & Wolfe Morrison, 2000). For example, the behavioural breach nuance is related to the actual breach itself, such as betrayal or disrespect, whilst the relational nuance is based on the type of relationship. This exploration of multidimensional breach of trust in the employment context has drawn on the literature regarding psychological contract breach and psychological contract violation (Morrison & Robinson, 1997). The psychological contract is the exchange agreement between an employee and employer (Rousseau, 1995) that is perceptual and idiosyncratic, based on perceived, rather than actual, promises and which can be transactional or relational (Morrison & Robinson, 1997). Robinson and Wolfe Morrison (2000) distinguished psychological contract breach, a cognitive evaluation of behaviour, from psychological contract violation which results from the emotions arising from the breach of the relationship. This is another way to conceptualise behavioural (psychological contract breach) versus relational (psychological contract violation) trust breach. Psychological contract breach (behavioural trust breach) is generally the trigger for psychological contract violation (relational trust breach; Henderson & O’Leary-Kelly, 2021). Psychological contract breach has been found to be negatively related to trust (Deery et al., 2006; Robinson, 1996).

Psychological contracts are unwritten agreements that develop between employees and leaders about work expectations and obligations (Rousseau, 1990) and that may exist regardless of the employee’s perception of the relationship with their leader. However, the employee–leader relationship plays a focal role in whether a psychological contract breach is perceived to have occurred, and this aligns with other literature near to the subject (Coyle-Shapiro & Conway, 2005; Henderson et al., 2008). Under Morrison and Robinson’s (1997) model of psychological contract breach, it is less likely that an employee in an established positive relationship with a leader will perceive a trust breach compared with an employee in a new relationship. Morrison and Robinson (1997) argued that an employee in an established relationship may be less vigilant to behaviour that may lead to a trust breach because they have a higher threshold for detecting such a breach. As a result, the employee is more likely to put the breach down to extenuating circumstances. However, when a trust breach is perceived in an established relationship, Morrison and Robinson (1997) also maintain that the resulting perceived psychological contract violation may be more severe because the breach is inconsistent with the social contract underlying the relationship. This is consistent with literature on psychological contract breach and employee–leader relationships (Doden et al., 2018; Restubog et al., 2009). Drawing on the literature of psychological contract breach and psychological contract violation (Morrison & Robinson, 1997), it is anticipated that trust breaches will have similar behavioural and relational nuances.

### Research aim and hypotheses

If “people leave managers, not jobs” (Elzinga, 2023; Kelly, 2022; Saiz, 2022), then it is important to understand the factors that create this phenomenon. The terms leader and manager have been used interchangeably in this paper to mean the same role of accountability over another person’s work and performance in an organisation. Although the literature is not extant, employee trust in leaders has been shown to mitigate intention to leave jobs (Rodwell et al., 2016; Erat, 2012). This evidence creates a rationale that understanding employee trust in leaders is important for ensuring organisational performance. This study aimed to understand the complexities of employee trust in leaders by exploring whether the relationship context or breach behaviour type will influence whether employees are likely to trust the leader in the future. Drawing on the literature regarding the role of the employee–leader relationship in psychological contract breach and psychological contract violation, it is hypothesised that:

- H1: The more established the relationship, the more likely the employee will trust the leader in the future following a trust breach.
- H2: Likelihood to trust the leader in future will be negatively influenced by all trust breaches, regardless of behavioural type.
- H3: If H1 is supported, and established relationships are more resilient to trust breach, then regardless of the behavioural nature of the breach there will be no interaction between relationship and behavioural breach type.

To test these hypotheses, participants were provided a hypothetical case of experiencing a behavioural breach of trust at work in the context of an employee–leader relationship. Then differences in response to likelihood of future trust by the behavioural treatment and the relationship treatment were compared.

## Method

### Participants

Study participants were all employed adults who have a leader to whom they report. Participant demographics are shown in Table 1. Although it was noted that there was skew to the younger ages of 18–34 and to those in the early-career stage, the analysis proceeded as generational difference may be a cognitive bias more than an influencing factor to the workplace (King et al., 2022).

**Table 1.** Participant demographics.

	Demographic characteristic	Frequency	Percentage
Gender	Female	250	52.0%
	Male	225	46.8%
	Gender non-specified	6	1.1%
Age	18–24	169	35.1%
	25–29	101	21.0%
	30–34	75	15.6%
	35–39	38	7.9%
	40–44	23	4.8%
	45–49	25	5.2%
	50–54	20	4.2%
	55–59	12	2.5%
	Over sixty	18	3.7%
Employment type	Full-time	346	71.9%
	Part-time	123	25.6%
	Contract or contingent worker	12	2.5%
Employment length	0–5 years	327	68.0%
	6–10 years	67	13.9%
	11–15 years	41	8.5%
	16–25 years	28	5.8%
	26–40 years	14	2.9%
	40+ years	4	0.8%
Industry	Agriculture	5	1.0%
	Mining	2	0.4%
	Manufacturing	29	6.0%
	Electricity, gas, water, and waste services	6	1.2%
	Construction	7	1.5%
	Wholesale trade	3	0.6%
	Retail trade	27	5.6%
	Accommodation and food services	24	5.0%
	Transport, postal and warehousing	16	3.3%
	Information media and telecommunications	44	9.1%
	Professional, scientific, and technical services	58	12.1%
	Administrative and support services	34	7.1%
	Public administration and safety	26	5.4%
	Education and training	33	6.9%
	Health care and social assistance	112	23.3%
	Arts and recreation services	10	2.1%
	Other services	45	9.4%

*N* = 481.

### Procedure

Ethics approval was obtained prior to commencement of the study. Participant recruitment was conducted in two parts. First, a sample was recruited via email and social media snowballing through researcher and participants’ communication networks. A second sample was recruited through a research panel via the data collection agency Prolific. Prolific is a platform to connect academic researchers and participants associated with the University of Oxford (Palan & Schitter, 2018). Consent was provided by submitting the online questionnaire. The rationale for the two samples was to ensure wider sample demographics and increased sample size.

For those who were recruited via email and social media snowballing, the advertisement outlined the nature of the study and invited voluntary participation. Consent was given by commencing the online questionnaire, and this was outlined in the Plain Language Statement and Consent Form. The advertisement outlined the nature of the study and invited voluntary participation. For those recruited via Prolific, participants were given the same Plain Language Statement and Consent Form prior to commencing the online questionnaire. All participants were screened by asking if they were currently employed and reported to a leader.

### Data handling

Data were screened through IBM SPSS Statistics Version 27 (IBM SPSS Statistics, 2020) to assess the accuracy of input, missing values, univariate outliers, linearity, and normality. After 96 cases were removed due to substantial missingness in the data, the total number of participants was 481. The sample size was considered appropriate for the one-way and two-way ANOVA and regression and moderation analyses (Schönbrodt & Perugini, 2013). To ensure comparability between those who received the survey through the snowball sampling technique and those who completed the survey via the research panel, a series of chi-square tests were conducted on Age and Gender. No significant differences between the participant groups were found, and therefore the samples’ recruitment source was not suspected to influence the results.

### Measures

#### Breach vignettes

The vignettes were short stories about a hypothetical situation presented to participants about themselves in the context of the workplace. The vignette stories were based on a qualitative analysis of employee trust in leaders (Fischer and Walker, 2022). Kähkönen et al. (2021) noted the need for greater qualitative study utilisation as a basis for future research in their recent systematic review. Fischer and Walker (2022) used critical incident technique in interviews with 38 employees and a grounded theory approach via reflexive and coding thematic analysis to interpret the results. Employing an inductive approach, Fischer and Walker (2022) found employee trust in leaders consisted of several relationship factors and explicit behavioural components. One member of the research team wrote the vignette stories. Each story was derived from isolating each of the behavioural components and placing them within different relationship contexts. Prior to commencement of the study, another member of the research team reviewed the vignettes to ensure readability and adherence to the qualitative research findings (Fischer and Walker, 2022).

The two independent variables tested were employee–leader relationship and behavioural trust breach. Employee–leader relationship had three treatments: no relationship, new relationship, and established relationship. The rationale and language used for the three treatments was based on the qualitative analysis findings (Fischer and Walker, 2022) about exposure (e.g., time spent and amount of interaction in the relationship), level of rapport and understanding in the relationship, amount of sameness (e.g., perceived similarity between the employee and leader). Regarding the behavioural breaches, there were eight treatments based on the trustworthy behaviours outlined by Fischer and Walker (2022). Each vignette comprised one relationship context setting and one behavioural breach scenario. An example vignette is below.

Last month, a new leader joined your team. Although you haven’t had much time to get to know them, you have learned about their career and education background and talked a bit about some common interests. You have had three catch up meetings since the new leader began in the role and you feel like you are just getting to know them.

Right before your fourth catch up is scheduled to occur, your leader says something condescending to you in your one-on-one meeting. They imply that your opinion is less significant based on your previous career experience and your place in the organisation.

The independent variable treatment descriptions are shown in Table 2. Each participant was randomly assigned one vignette to complete via the survey platform software. In total, there were 24 possible vignette options. Table 2 shows frequencies relating to how many participants received each treatment.

**Table 2.** Independent variables’ treatment frequencies and means.

Independent variable	Treatment	Frequency	Percent	Means
Relationship	No relationship	160	33.3%	1.90
	New relationship	160	33.3%	1.91
	Established relationship	161	33.3%	2.37
Behavioural breach	Lying	60	12.5%	1.75
	Unethical behaviour (fraud)	60	12.5%	2.07
	Betraying confidentiality	61	12.7%	2.07
	Accidentally omitting information	60	12.5%	1.82
	Betraying agreements	60	12.5%	2.18
	Denying request for support	60	12.5%	2.20
	Condescension	60	12.5%	2.55
	Public belittling	60	12.5%	2.15

Total *N* = 481.

#### Likelihood to trust

Trust propensity theory presented a challenge for this research (Alarcon et al., 2016; Mayer et al., 1995; McKnight et al., 1998). Given the trustor–trustee relationship develops in time, and the trustor becomes more familiar with the trustee based on actual experience (Levin et al., 2004), the decision was made to assess the participants’ actual experience of the vignette. Trust measurement is varied (Dirks & Ferrin, 2002; Fischer et al., 2023; McEvily & Tortoriello, 2011), and there does not appear to be a reliable and available measure for likelihood to trust in the future in the literature. Therefore, after reading the vignette, likelihood to trust was measured by asking participants *what is the likelihood that you would trust this leader in the future*. The rating scale was a five-point Likert scale (1 = Strongly Distrust and 5 = Strongly Trust).

### Analysis

The analysis of variance (ANOVA) was selected to determine the impact of relationship and behavioural breaches on likelihood of future trust in the leader. The first step of the analysis was to assess if there were differences between each independent variable treatment conditions using one-way ANOVA. Next, a two-way ANOVA was conducted to determine if there were any interactions between independent variable treatment conditions. Finally, Tukey’s Honest Significant Difference (HSD) test was conducted after each one-way and two-way ANOVA. Tukey’s HSD showed which treatment group’s means were different when compared with another (Salkind, 2010).

## Results

### One-way ANOVA

Two one-way ANOVAs were conducted to investigate whether the independent variables had any significant differences in the means of likelihood of future trust ratings. The first one-way ANOVA explored the effect of the three different relationship conditions (see Table 3). A post-hoc Tukey HSD test showed that the established relationship group (mean = 2.37) differed significantly at *p* < .001 from both the no relationship (mean = 1.90) and new relationship (mean = 1.91) groups. There was no significant difference found between the no relationship or new relationship groups. Therefore, H1 was supported.

**Table 3.** One-way analysis of variance summary table for relationship type on participants’ perceptions of future trust.

Source	*df*	*SS*	*MS*	*F*	*p*
Between-group	2	23.318	11.659	12.017	>0.001
Within-group	477	462.807	0.970		
Total	479	486.125			

The second one-way ANOVA repeated the analysis but focused on the differences of behavioural breach type (see Table 4). A post-hoc Tukey HSD test was again applied to test which behavioural breach types were significantly different to one another. All treatments showed a negative influence on likelihood to trust in future in the mean score (see Table 2), which supported H2. All treatment means were below an average of 3 (or “unsure” in the 5-point Likert rating scale) and suggests that regardless of relationship length participants disagree-strongly disagree that they will trust the leader in the future. Interestingly, the test showed that behavioural breaches a) *leader lying to the employee* (*m* = 1.75), c) *leader betraying confidentiality* (*m* = 1.82), and h) *leader belittling employee publicly* (*m* = 1.77), were significantly different to behavioural breach f) *leader denying the employee support* (*m* = 2.56). No further significant differences emerged between behavioural breach types. Lying, betraying confidentiality and belittling employees were the lowest rated behaviours in terms of future trust in the leader post breach.

**Table 4.** One-way analysis of variance summary table for behaviour breach type on participants’ perceptions of future trust.

Source	*df*	*SS*	*MS*	*F*	*p*
Between-group	7	31.200	4.457	4.634	>0.001
Within-group	473	454.929	0.962		
Total	480	486.129			

### Two-way ANOVA

A two-way ANOVA was conducted to determine if there were any interaction effects of relationship and behavioural breach type. In support of H3, no interaction effects were found, and therefore no post-hoc tests were conducted (see Table 5).

**Table 5.** Two-way analysis of variance summary table for relationship and behaviour breach type on participants’ perceptions of future trust.

Source	*df*	*SS*	*MS*	*F*	*p*
Corrected Model	23	72.508*	3.153	3.483	>0.001
Intercept	1	2051.120	2051.130	2266.244	>0.001
Relationship	2	22.928	11.464	12.666	>0.001
Behaviour	7	30.789	4.398	4.860	>0.001
Relationship*Behaviour	14	18.303	1.307	1.444	0.129
Error	457	413.621	.905		
Total	481	2532.000			
Corrected Total	480	486.129			

*R Squared = .149 (Adjusted R Squared = .106).

## Discussion

The study aimed to understand how the relationship context or breach behaviour type would influence whether employees are likely to trust the leader in the future. The findings of this study reinforce the role of the relationship in employee trust in leaders (Gustafsson et al., 2020). There were no statistically significant differences found in responses from the 1) no employee relationship with the leader or the 2) new employee relationship with the leader groups and the means were nearly identical. However, statistical differences were found with the 1) no and 2) new employee relationship with the leader groups and the group who responded to vignettes about a leader where there was 3) an established relationship. This suggests that even in hypothetical employee–leader relationships, participants were more inclined to trust their leader in the future after a trust breach when there was a perceived established relationship.

The findings of this study also reinforce the behavioural dimension of employee trust in leaders. The behavioural breaches that emerged as the lowest trust ratings were leader *lying to*, *betraying confidentiality of*, and *publicly belittling* the employee. Those three behavioural breach treatments were also significantly different from leader *denying the employee support*. The behavioural breaches of leader *unethical behaviour (fraud)*, *accidentally omitting information*, *betraying agreements*, *denying request for support*, and *condescension* were not found to be statistically significant from one another, suggesting that the type of behavioural breach matters in some cases and can influence perceptions of future trust.

There was no interaction found between relationship and behavioural breach type. Therefore, according to this study’s findings and consistent with Morrison and Robinson (1997), regardless of most behavioural breach types, established employee–leader relationships are more likely to be resilient to violation than when the relationship is new or absent between the employee and leader. This finding is also consistent with Gustafsson et al.’s (2020) theory of trust preservation after breach. Cognitive bridging, emotional embodying, and inclusive enacting (Gustafsson et al., 2020) are psychological processes that occur through the interpersonal relationship between employee and leader.

### Psychological safety compromised by extreme breach behaviour

The behaviours of leader lying to, betraying confidentiality of, and publicly belittling the employee are extreme and severe. It is possible, that even in a hypothetical situation, the impact of such negative behaviours may compromise psychological safety of the employee regardless of the employee–leader relationship. Psychological safety at work is a shared belief across individual employees in a group that it is safe for interpersonal risk taking without fear of negative consequences to self-esteem, status, or career (Edmondson & Lei, 2014). Employee psychological safety in the context of the employee–leader relationship is critical for trust in the workplace, and without it trust cannot be developed or maintained (Fischer and Walker, 2022). Psychological safety is part of the psychological contract of trust (Fischer and Walker, 2022) that influences employee and leaders’ behaviours and feelings towards the other (Robinson, 1996; Rousseau, 1995). Teams need psychological safety to perform, as work requires team members to communicate, ask questions and share ideas and concerns with colleagues and stakeholders across all levels (Newman et al., 2017). If psychological safety is compromised, then communication, learning, and innovation can be stifled (Baer & Frese, 2002) and engagement decreased (Ge, 2020). This study’s findings reinforce that when trust is breached by a behaviour that possibly damages the employee’s psychological safety, it may be harder to re-establish that trust, more so than other behavioural breaches that may be perceived by the employee as less risky to psychological safety.

### Implications

This study showed how the breach-type context can influence future employee trust in the leader. This study’s findings uphold the importance of the employee–leader relationship (Carter et al., 2012) and suggest that the stronger the relationship between the leader and employee the more likely trust can be restored after a breach. Leaders who put effort into building strong, positive relationships can benefit from this. However, there are some cases when the relationship cannot buffer the effects of poor behaviour. If the leader lies to their employee, betrays their confidence, or publicly belittles the person, then future trust is less likely to be restored. Therefore, leaders must be mindful of the negative impact of these behaviours on employee trust and their psychological safety.

### Limitations

Although the findings of this study are promising, there are some important limitations in the sample to note. The use of the two recruitment methods may have influenced the findings. Some participants were recruited via email and social media snowballing through researcher and participants’ communication networks. The second sample was recruited through a research panel via the data collection agency Prolific. Work location was not asked of participants, so it is not possible to determine if this variable influenced outcomes. The potential issue regarding recruitment strategy differences was partially dealt with by exploring comparability through a series of chi-square tests on Age and Gender where no significant differences between the participant groups were found.

The study randomly allocated participants to one of the vignettes. Although random allocation has the advantage of reducing bias (Hopp, 2014), it may have resulted in the allocation of participants to a relationship about which they had no experience. Over half of the participants were aged under 30 and almost two-thirds reported having been in their role for <5 years. These participants may not have experienced being in an established employee–leader relationship. If these participants were assigned to the established relationship condition, their consideration of the vignette could have been hypothetical, which may have affected the accuracy of their response. This is an artificial setting and therefore the findings may be questioned for reliability in the real work world. The outcomes of this study make a strong case for replication using real work examples. Finally, the study did not account for the effect of participant’s previous experiences. Measuring likelihood to trust in future with one item may have biased the results, although no measure of likelihood to trust in future could be found in the literature (Dirks & Ferrin, 2002; Fischer et al., 2023; McEvily & Tortoriello, 2011). Established theories regarding trust (Schoorman et al., 2007), psychological contract breach (Morrison & Robinson, 1997) and the literature relating to breach of trust and leader-member exchange (e.g., Doden et al., 2018) all suggest that employee past experiences of employers, leaders and breaches of trust may impact their future likelihood to trust. For example, employees who have previously worked with a leader who breached their trust in a particular way are more likely to be vigilant to future similar circumstances (Morrison & Robinson, 1997). Taking participants’ past experiences and recent context into account would require a more complex study design involving qualitative analysis of the reasons for participants’ choices.

### Future research

Future research could consider replicating this study with other populations to determine generalisability. Researchers could also consider replicating the study using employees’ actual experiences at work where trust breach has occurred in similar relationship and behavioural breach contexts. The study’s design was cross-sectional. This brought advantages, namely the ability to consider a wide range of scenarios with a sample size that enabled the proposed analyses to be undertaken. However, participants considering the vignettes had to “imagine” themselves in an employee–leader relationship based on comparatively little information. It is possible that participants’ future trust intention in these hypothetical scenarios is quite different from their intention in real-life scenarios where the information on which they make their decisions would be richer. A longitudinal study would resolve this issue. Future research should also consider participants’ past experiences of trust breach by leader at work to explore if that influences the research outcomes.

## Conclusion

Employee trust in leaders is essential for organisational performance (Oh, 2019; Vanhala & Tzafrir, 2021), and improving employee trust in leaders may prevent the adage of “people leave managers, not jobs” (Elzinga, 2023; Kelly, 2022; Saiz, 2022). This study reinforces that employee trust in leaders is multidimensional, and that there is a behavioural and relational element. Findings demonstrated that the stronger the relationship, the more likely the employee is to trust the leader in the future after a trust breach. The study also demonstrated that not all behavioural breaches are created equal. When a leader lies, betrays confidentiality, or publicly belittles the employee, trust is least likely to recover from such a breach, regardless of the relationship, and that this is possibly due to impact on employee psychological safety. Leaders will benefit from investing in their relationships with employees and must exercise caution when demonstrating untrustworthy behaviour if they wish the employee to trust them in the future.

## Data Availability

Due to the nature of this research, participants of this study did not agree for their data to be shared publicly, so supporting data is not available.
